# Efficacy of Antioxidant Supplementation on Conventional and Advanced Sperm Function Tests in Patients with Idiopathic Male Infertility

**DOI:** 10.3390/antiox9030219

**Published:** 2020-03-06

**Authors:** Mohamed Arafa, Ashok Agarwal, Ahmad Majzoub, Manesh Kumar Panner Selvam, Saradha Baskaran, Ralf Henkel, Haitham Elbardisi

**Affiliations:** 1 Male Infertility Unit, Urology Department, Hamad General Hospital, 00974 Doha, Qatar; dr.amajzoub@gmail.com (A.M.); elbardisi@hotmail.com (H.E.); 2 Urology Department, Weill Cornell Medical-Qatar, 00974 Doha, Qatar; 3 American Center for Reproductive Medicine, Cleveland Clinic, Cleveland, OH 44195, USA; agarwaa@ccf.org (A.A.); pannerm@ccf.org (M.K.P.S.); saradhabaskaran@gmail.com (S.B.); rhenkel@uwc.ac.za (R.H.); 4 Andrology Department, Cairo University, 11562 Cairo, Egypt; 5 Department of Medical Bioscience, University of the Western Cape, 7535 Bellville, South Africa

**Keywords:** idiopathic male infertility, unexplained male infertility, antioxidants, oxidation-reduction potential, sperm DNA fragmentation

## Abstract

Antioxidants are used in the empirical treatment of infertile men. The aim of this study was to evaluate the effects of antioxidant therapy on conventional semen parameters and advanced sperm function tests in men seeking fertility treatment. A total of 148 infertile men of unknown etiology were divided into idiopathic (*n* = 119) and unexplained male infertility (UMI; *n* = 29). All participants were treated with the antioxidant supplement ‘FH PRO for Men’ for a period of three months. Compared with pretreatment results, there was a significant improvement in conventional semen parameters including sperm concentration, total and progressive motility and normal morphology, and seminal oxidation reduction potential (ORP), and sperm DNA fragmentation (SDF) in idiopathic infertile men. The changes were more prominent in idiopathic infertile men positive for ORP and SDF. UMI patients showed an improvement in progressive motility, ORP, and SDF after antioxidant treatment. Statistical analysis revealed that the efficacy of FH PRO for Men was significant in idiopathic male infertility compared with UMI. Treatment of idiopathic male infertility patients with the FH PRO for Men antioxidant regimen for three months resulted in a significant improvement in conventional semen parameters and sperm function. Therefore, FH PRO for Men offers promise for the medical treatment of idiopathic male infertility.

## 1. Introduction

Infertility is a global health problem affecting 15% of couples world-wide, with the male factor contributing to almost half of the reported cases [1,2]. Despite the extensive research and advances in male reproductive health, a significant proportion of male infertility cases are idiopathic [3]. Idiopathic male infertility is characterized by the presence of abnormal semen parameters without a discernible cause and absence of female factor infertility [4]. On the other hand, men exhibiting infertility owing to an unknown origin with normal semen parameters and having female factor ruled out are categorized as unexplained male infertility (UMI) [5]. Overwhelming evidence suggests the central role of oxidative stress in the etiology of male infertility [6,7,8], and about 30–80% of infertile men have been reported to have elevated levels of seminal reactive oxygen species (ROS) [4]. Recently, the term “male oxidative stress infertility” (MOSI) has been coined “as a novel descriptor for infertile men with abnormal semen characteristics and oxidative stress” and has been reported to influence 37.2 million men with idiopathic infertility [4].

Oxidative stress prevails when the levels of ROS exceeds the scavenging potential of antioxidant defense system. ROS have a dual role in male fertility as well as infertility [9]. While it is true that low levels of ROS are crucial for normal sperm functions, high levels have been reported to cause peroxidative damage to spermatozoa, thereby jeopardizing their fertilizing ability [10]. Hence, maintaining the levels of ROS within physiological levels is crucial for conserving the structural and functional integrity of spermatozoa. Increased oxidative stress results in the alteration of semen characteristics by inducing lipid peroxidation of sperm plasma membrane, oxidation of crucial sperm proteins, and sperm DNA fragmentation (SDF), leading to male infertility [11,12].

With an increasing acknowledgment of the role of oxidative stress in the pathophysiology of male infertility, antioxidant supplementation is recommended as one of the treatment choices for idiopathic infertile men [4,13]. Oral supplementation of a single antioxidant or combination of antioxidants such as l-carnitine, l-acetyl carnitine, *N*-acetyl-cysteine, Coenzyme Q10, selenium, vitamin C, vitamin E, and lycopene has been reported to improve semen parameters and sperm DNA integrity in idiopathic infertile men [14,15,16,17,18,19,20]. Furthermore, multi-antioxidant supplementation is considered more effective for male fertility parameters owing to the synergetic effects of antioxidants [16,21,22]. A study conducted by Gharagozloo et al. investigated the efficacy of Fertilix^®^, an antioxidant formulation, using two established mouse models of oxidative stress (scrotal heating and *Gpx5* knockout mice) and reported protection of sperm DNA against oxidative damage and an increased pregnancy rate [22]. A recent systematic review involving 29 studies (19 randomized clinical trials and 10 prospective studies) examined the effect of oral antioxidant therapy on fertility outcomes and reported a positive effect of antioxidant supplementation on basic semen parameters, advanced sperm function, outcomes of assisted reproductive therapy (ART), and live birth rate [23]. Conversely, several studies failed to confirm any positive effect of antioxidant therapy or even reported a negative impact on male fertility [24,25,26]. Therefore, there is not yet a clear consensus regarding the clinical effectiveness of antioxidant therapy in male infertility. In light of the above, the present study was undertaken to evaluate the efficacy of antioxidant supplementation with ‘FH PRO for Men’ on conventional and advanced sperm function tests in men with idiopathic infertility and men with UMI.

## 2. Materials and Methods

### 2.1. Study Design and Ethics Statement

**Figure 1:**
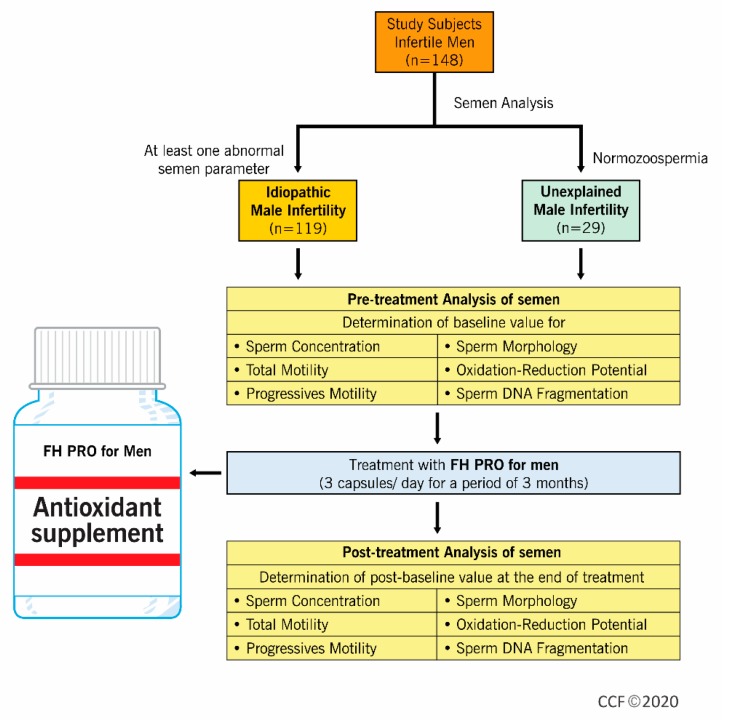
Flow diagram depicting the experimental design.

This prospective clinical trial was registered under linicaltrials.gov (NCT03464656) and conducted in the Male Infertility Unit of the Department of Urology, Hamad Medical Corporation, Doha, Qatar, in collaboration with the American Center for Reproductive Medicine, Cleveland Clinic, Cleveland, Ohio, from March 2018 to January 2019. A total of 148 infertile men were included in this study (Figure 1). All patients gave written consent before being recruited in the study. The Institutional Review Board (IRB) of Hamad Medical Corporation approved the study protocol (HMC-IRB #16351/16).

### 2.2. Study Subjects

Adult males presenting to the male infertility clinic were screened for inclusion and exclusion criteria before enrollment in the study. Infertile men (between 20 and 50 years of age) with unknown etiology and female infertility factor ruled out were included in the study. On the basis of semen analysis, subjects enrolled were categorized into idiopathic and UMI. The idiopathic infertility group included men with abnormal semen analysis defined as having at least one sperm parameter (sperm concentration >1 and ≤15 million per mL, total sperm motility ≤40%, or sperm morphology as evaluated by strict criteria with normal forms ≤4.0%). In the UMI group, infertile men with normal semen parameters (sperm concentration >15 million per mL, total sperm motility >40%, or sperm morphology as evaluated by strict criteria with normal forms >4.0%) were included [27]. Exclusion criteria included patients with azoospermia or with a sperm concentration <1 million per mL and those with leukocytospermia. In addition, patients having any identifiable cause for infertility such as clinical varicocele (grade 2 and higher), orchitis, epididymitis, cryptorchidism, genetic cause for infertility, or irradiation, as well as subjects who received chemotherapy treatment, clinically meaningful endocrinopathy defined as an endocrinopathy (which requires endocrine medications, for example, diabetes, thyroid disease, pituitary diseases, adrenal diseases, and so on), or abnormal hormonal profile (testosterone <10.4 nmol/L, luteinizing hormone (LH) <1 or > 9 IU/L, and/or follicle stimulating hormone (FSH) <1 or >19 IU/mL, elevated prolactin >407 mIU/L, elevated thyroid-stimulating hormone (TSH) >4.5 U/mL, elevated estrogen >275 pmol/L) were also excluded from the study. Patients receiving antioxidants in the past six months were excluded. Also, patients with dietary or social habits (subjects following any special diet including, but not limited to liquid, high or low protein, raw food, vegetarian, or vegan, among others, as well as consumption of more than one unit of alcohol daily, and history or current use of illegal or “recreational” drugs), as well as medical conditions (such as known HIV infection, malignancy, and renal or hepatic failure) that may impact oxidative stress, were also excluded. Twenty-one patients out of 171 in total were excluded based on the above criteria. Of the remaining 150, only 2 dropped out from the study, while remaining 148 completed the trial.

### 2.3. Study Protocol

A physician specializing in male infertility obtained a full medical history from each study participant, and performed a physical examination (general and local genital) on each participant. Standard semen analysis, SDF, and seminal oxidation-reduction potential (ORP) were evaluated at the beginning of the study. Patients were prescribed to take three capsules of the antioxidant formula “FH PRO for Men” (Fairhaven Health LLC, Bellingham, WA, USA) twice a day for three months, which provides the following amounts of these nutrients per day: vitamin A (as beta-carotene): 5000 IU, vitamin C: 120 mg, vitamin D3: 1200 IU, vitamin E (as mixed tocopherols): 200 IU, vitamin K: 80 µg, thiamin: 3 mg, riboflavin: 3.4 mg, niacin: 20 mg, vitamin B6: 25 mg, folate: 800 µg, vitamin B12: 1000 µg, biotin: 600 µg, pantothenic acid: 20 mg, iodine: 150 µg, zinc: 30 mg, selenium: 140 µg, copper: 1 mg, manganese: 2 mg, chromium: 120 µg, molybdenum: 75 µg, l-carnitine tartrate: 2000 mg, l-arginine: 350 mg, CoQ10: 200 mg, *N*-acetyl l-cysteine: 200 mg, grapeseed extract: 20 mg, lycopene: 10 mg, and benfotiamine: 1 mg. Thorough monitoring of the patients’ compliance with the medication was done through bi-weekly phone call follow-up. After finishing the three months of treatment, patients were examined again, and semen analysis, SDF, and ORP were reassessed.

### 2.4. Semen Analysis

After complete liquefaction, each sample was evaluated for macroscopic parameters such as color, pH, ejaculate volume, and viscosity. An aliquot of the sample was examined for sperm concentration, total sperm count, total and progressive motility, and sperm morphology using the WHO Fifth Edition guidelines (WHO, 2010) [27]. Semen analysis was done manually using a hemocytometer. Sperm motility was assessed and categorized as progressive or non-progressive. Morphology was assessed by a single experienced technician using the Diff–Quik staining protocol and evaluated according to strict criteria for normal sperm morphology with 4% normal morphology as a cut-off (WHO, 2010). Patients were classified according to WHO (2010) criteria into ‘oligozoospermic’, ‘asthenozoospermic’, and/or ‘teratozoospermic’.

### 2.5. Sperm DNA Fragmentation (SDF) Assessment

SDF was evaluated using the Halosperm kit from Halotech DNA, S.L. (Madrid, Spain) as per the manufacturer’s instructions. The method is based on the sperm chromatin dispersion test [28]. In brief, unfixed spermatozoa were immersed in an inert agarose microgel on a pretreated slide. An initial acid treatment denatures DNA in the spermatozoa with fragmented DNA. The lysing solution removes most of the nuclear proteins, and in the absence of large DNA breaks, produces nucleoids with large halos of spreading DNA loops, emerging from a central core. Spermatozoa with fragmented DNA do not produce or show a very small dispersion halo. Both positive and negative controls were included. For the positive control (sperm with halo), the acid denaturation (0.08 N HCl) step was omitted. For the negative control (sperm without halo), after removing the coverslip, 10 µL of undiluted denaturation solution was applied and a cover slip was gently placed without pressure and left for 5 min. A minimum of 500 spermatozoa were scored and reported as percentage of sperm with fragmented DNA. A cut-off value of 30% was used to differentiate normal from high SDF [29]. Accordingly, patients were classified into high (>30% SDF) and low (≤30% SDF) groups.

### 2.6. Oxidative Stress Assessment

Oxidative stress was assessed by measuring the static ORP of neat liquefied semen samples using the MiOXSYS^TM^ (Aytu Bioscience, Inc., Englewood, CO, USA). This is a galvanostatic measure of the electron transfer from reductants (antioxidants) to oxidants under a steady low voltage reducing current. Thus, it provides an aggregate measure of all current oxidant and antioxidant activity in a sample. Higher ORP values (mV) indicate a higher oxidant activity relative to the antioxidant activity and, therefore, a greater state of oxidative stress. Each sample was run in duplicate. Briefly, a 30 µL aliquot of liquefied semen was loaded on a test sensor that had been pre-inserted into the MiOXSYS analyzer (Aytu Bioscience, Englewood, CO, USA). No specific preparation of the semen samples is needed to perform the test. To control for differences in sperm count, ORP values were normalized by dividing the ORP with the sperm concentration (×10^6^/mL) and are represented as mV/10^6^ sperm/mL [30]. A cut-off value of 1.34 mV/10^6^/mL was used to differentiate normal from abnormal semen parameters [31]. According to this classification, patients were grouped into high ORP (>1.34 mV/10^6^ sperm/mL) and low ORP (≤1.34 mV/10^6^ sperm/mL).

### 2.7. Statistical Analysis

Statistical analysis was performed using MedCalc statistical software version 19.1 (MedCalc Software bv, Ostend, Belgium). After testing for normal distribution using the Chi-squared test, parametric (Pearson’s correlation, paired samples t-test) or non-parametric (Spearman’s Rank correlation, Wilcoxon test) tests were employed. In addition, the McNemar test was used to test the difference between paired proportions. Qualitative and quantitative measurements were summarized using frequency with percentage and mean ± SD. A *p*-value less than 0.05 was considered statistically significant.

## 3. Results

The study subjects reported no adverse effect of antioxidant supplement during or after six months of the trial. Of the 148 patients who completed this clinical trial, 102 patients were diagnosed with primary infertility and 46 of secondary infertility with a mean duration of infertility of 5.8 ± 4.3 years. The mean age of study participants was 35.9 ± 0.5 years. A total of 119 of the 148 subjects included in the study showed abnormal semen parameters, while 29 were normozoospermic with UMI. Table 1 compares all semen parameters of both groups, except sperm vitality as it was evaluated only in patients with <40% total motile sperm.

### 3.1. Effect of Antioxidant Supplementation on Sperm Parameters

#### 3.1.1. Idiopathic Infertility Group

Table 2 summarizes the clinical data and investigations of all 119 infertile patients with abnormal semen parameters pre- and post-treatment with FH PRO for Men. Supplementation of idiopathic infertile men with FH PRO showed a significant decrease in seminal ORP and SDF levels (Table 2). Post-treatment showed significant (*p* < 0.05) improvements compared with pre-treatment results with regards to all parameters investigated, except for semen volume and sperm viability. Further, the sub-category of idiopathic infertile men (*n* = 108) having high levels of ORP (≥ 1.34 mV/10^6^ sperm/mL) after antioxidant treatment showed a significant change in their semen parameters (sperm concentration, total motility, progressive motility, and normal sperm morphology) (Table 3). We also noticed a significant difference in the pre- and post-treatment semen parameters of the idiopathic infertile men (*n* = 51) having high levels of SDF (>30%) (Table 4). Altogether, supplementation of idiopathic infertile men having both high levels of ORP and SDF with FH PRO for Men showed a significant improvement in the semen parameters (Table 5).

#### 3.1.2. Unexplained Male Infertility Group

**Figure 2:**
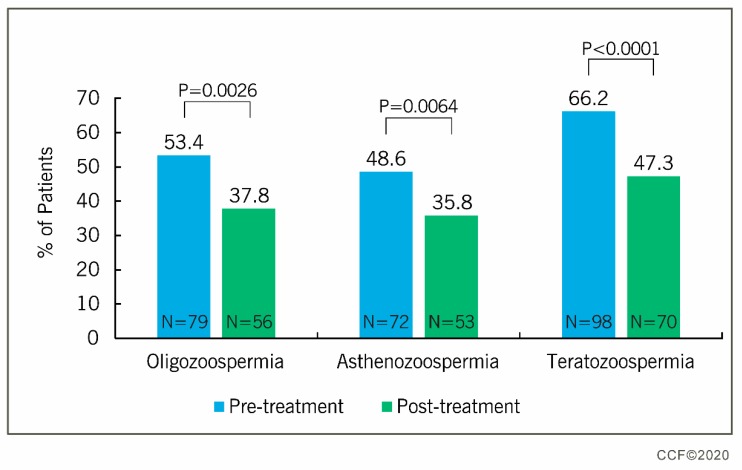
Outcome of FH PRO treatment on infertile men (*n* = 119) with oligozoospermia (sperm concentration <15 × 10^6^/mL), asthenozoospermia (total motility <40%), and teratozoospermia (normal sperm morphology <4%). McNeamar test was used to calculate the percent change.

Table 6 depicts the summary results of before and after the antioxidant supplementation for the 29 normozoospermic men with unexplained infertility. Comparing the men with UMI against idiopathic infertile men in response to treatment with FH PRO for Men revealed that idiopathic infertile men showed significant differences in their semen parameters (sperm concentration, total motility, progressive motility, and normal sperm morphology), while in men with UMI only progressive motility improved significantly (Table 2 and Table 6). Furthermore, the treatment of idiopathic infertile men (oligo-, astheno-, and teratozoospermia) with FH PRO for Men resulted in the significant reduction in the percentage of oligozoospermic (53.4% vs. 37.8%), asthenozoospermic (48.6% vs. 35.8%), and teratozoospermic (66.2% vs. 47.3%) patients (Figure 2 and Supplementary Table S1).

### 3.2. Effect of Antioxidant Supplementation on Seminal ORP

#### 3.2.1. Idiopathic Infertility Group

Out of the 119 patients with idiopathic infertility, 108 had high initial ORP (90.8%) while only 74 (62.2%) had high ORP after the treatment. After antioxidant treatment, the seminal ORP levels significantly decreased (*p* < 0.05) in men with idiopathic infertility (Table 2). Further, in the sub-category of idiopathic infertile men (*n* = 108) having high levels of ORP (≥1.34 mV/10^6^ sperm/mL), the mean ORP levels were 13.5 ± 17.2 mV/10^6^ sperm/mL before the treatment. After treatment with FH PRO for Men, the ORP levels decreased to 8.0 ± 16.4 mV/10^6^ sperm/mL (Table 3). A similar decrease in the seminal ORP levels was noticed in post-treatment idiopathic infertile men (*n* = 51) having high levels of SDF (>30%) (Table 4). Further, supplementation of idiopathic infertile men (*n* = 46) having high levels of both ORP and SDF with FH PRO for Men showed a significant reduction in the seminal ORP levels from 6.7 ± 6.6 mV/10^6^ sperm/mL to 3.7 ± 8.9 mV/10^6^ sperm/mL (Table 5).

#### 3.2.2. Unexplained Male Infertility Group

In men with UMI (*n* = 29), 14 (50.0%) had high ORP values before supplementation with FH PRO for Men; following supplementation, only eight patients (28.6%) showed high ORP levels (Table 7). The results further showed that treatment of UMI patients with FH PRO for Men resulted in a significant improvement in seminal ORP (1.6 ± 1.1 mV/10^6^ sperm/mL vs. 1.1 ± 0.9 mV/10^6^ sperm/mL; *p* < 0.05) (Table 6). Considering both groups (men with idiopathic male infertility and UMI) separately, the McNemar test showed a positive effect of FH PRO for Men by reducing seminal OPR in idiopathic infertile patients. The percentage of patients with high ORP levels decreased from 90.8% to 63.0% post-treatment, whereas in men with UMI, there was no significant improvement after treatment (Table 7).

### 3.3. Effect of Antioxidant Supplementation on SDF

#### 3.3.1. Idiopathic Infertility Group

SDF was assessed in only 112 patients (83 men with idiopathic infertility; 29 men with UMI) having sperm concentration >5 × 10^6^/mL. Supplementation with FH PRO for Men resulted in a significant decrease (*p* < 0.05) in SDF levels (43.5% ± 22.8 to 34.3% ± 19.4) in men with idiopathic infertility (Table 2). Also, idiopathic infertility patients (*n* = 67) having high levels of ORP (≥1.34 mV/10^6^ sperm/mL) showed a reduction in SDF levels after the treatment (Table 3). Similar effects on SDF levels were observed in post-treatment idiopathic infertile men (*n* = 47) with high initial levels of SDF (>30%) (Table 4). Further, supplementation of idiopathic infertile men (*n* = 42) having high levels of both ORP and SDF with FH PRO for Men showed a significant reduction in the SDF levels from 56.3% ± 16.5% to 44.0% ± 20.3% (Table 5).

#### 3.3.2. Unexplained Male Infertility Group

Unexplained infertility patients (*n* = 29) showed a decrease (*p* < 0.05) in SDF levels following treatment with FH PRO for Men. However, the pre- and post-treatment SDF levels remained below the cut-off value of 30% (Table 6). The McNemar test revealed that, after antioxidant treatment, the SDF levels in idiopathic infertile men reduced below the cut-off value of 30%. Whereas, in normozoospermic infertile patients, the effect of FH PRO for Men on SDF levels was not significant (Table 7).

## 4. Discussion

Oxidative stress has been implicated in the pathophysiology of male infertility and the associated increase in free radicals, inducing sperm dysfunction and high SDF [12]. Moreover, imbalance in the levels of ROS and antioxidant enzymes coupled with increased peroxidative damage to sperm plasma membrane and DNA have been frequently reported in male infertility of unknown origin [4,32,33,34]. In this context, antioxidant supplementation has been recommended as one of the treatment choices for men with idiopathic infertility [4,13]. Though several studies have reported beneficial effects of antioxidant supplementation in idiopathic infertile men [18,19,20,35,36], some reported the negative impacts of antioxidant therapy on male infertility [24,25,26]. Hence, there is not yet consensus on the clinical effectiveness of antioxidant therapy in male infertility. The present study was conducted to evaluate the efficacy of FH PRO for Men treatment in idiopathic infertile men (non-normozoospermic) and unexplained (normozoospermic) male infertility. We compared the pre- and post-treatment levels of conventional sperm parameters and advance sperm function tests in each group as well as between the groups to assess the efficacy as well as the response to the antioxidant supplementation. For the first time, this study sheds light on the possible treatment of idiopathic male infertility and UMI using an antioxidant formulation.

An antioxidant formulation is considered to be beneficial for infertile men when it is able to bring about a positive change in basic semen parameters such as sperm concentration, total sperm motility, progressive motility, and normal sperm morphology as per 2010 WHO Fifth Edition criteria. Several clinical trials have reported an improvement in semen parameters after antioxidant treatment [16,18,20,37,38,39]. In the current trial, men with idiopathic infertility showed an improvement in semen parameters such as sperm concentration, total motility, progressive motility, and normal sperm morphology after treatment with FH PRO for Men, a multi-ingredient dietary supplement containing antioxidants, vitamins, and minerals in adequate dosages to produce a positive synergistic effect on sperm parameters. The inclusion of spermatogenic components such as l-arginine and zinc in the recommended dosage improved the sperm concentration significantly in idiopathic infertile men. However, the changes were more prominent in idiopathic infertile men with high levels of oxidative stress and sperm DNA damage. Agarwal et al. 2019 reported that proteins such as PRKAR1A, PRKAR2A, PRKACA, and LDHC associated with the CREM signaling pathway were activated in idiopathic infertile men after treatment with FH PRO for Men [40]. Proper functioning of the CREM signaling pathway is essential for the differentiation of spermatids [41]. Sperm maturation proteins such as CLU and TPP2 were also reported to be overexpressed in idiopathic infertile men following treatment with antioxidant formulation [40]. Therefore, the significant increase in semen parameters of idiopathic infertile men following treatment could be because of the activation of proteins associated with spermatogenesis at the molecular level by the components present in FH PRO for Men. Furthermore, the increase in the total and progressive motility of sperm after antioxidant treatment may be because of the action of l-carnitine tartrate, which is a source of energy production in matured spermatozoa [42]. This is supported by the proteomic and bioinformatic results, demonstrating the activation of the transcription factor PPARGC1A associated with sperm motility and capacitation [40]. In the case of normozoospermic men with UMI, FH PRO for Men did not show a significant change in sperm concentration, total motility, and sperm normal morphology, but a significant increase was noticed in the progressive motility. Overall, the improvement in sperm parameters of infertile men with abnormal semen parameters clearly indicates that FH PRO for Men has a higher efficacy in men with idiopathic infertility. There was a significant decrease in the number of patients categorized as oligozoospermic, asthenozoopspermic, and teratozoospermic after antioxidant treatment, which reflects an improvement in sperm quality and function owing to supplementation with antioxidants. These findings are in agreement with the changes observed previously by Agarawal et al. at the subcellular level, such as overexpression of sperm binding and fertilization proteins after treatment with FH PRO for Men [40].

In semen, a balance between ROS and antioxidant systems establishes redox homeostasis, which is essential for the normal functioning of spermatozoa. Excessive production of ROS decreases the concentration of antioxidants and results in seminal oxidative stress, which is considered as one of the major factors contributing to male infertility. Conventionally available techniques can either detect oxidative stress directly or indirectly, but do not provide the complete measure of seminal redox status. Recently, ORP has been used as a tool to evaluate the seminal oxidative stress, which provides a comprehensive analysis of the seminal oxidant and reductant levels [20,43]. Furthermore, increased levels of ORP were also found to have a negative impact on the sperm vitality and mitochondrial membrane potential [44,45]. In the current study, we noticed a significant decrease in seminal ORP levels in infertile men after antioxidant treatment. Idiopathic infertile men positive for ORP and SDF showed a drastic decrease in the ORP levels after antioxidant treatment. A decrease in ORP levels was also observed in UMI patients, however, this decrease in UMI patients did not reach statistical significance (Table 7). The antioxidant formulation used in the current study includes l-carnitine tartrate, arginine, and Co-Q10, which are known to improve semen quality [23,46,47]. At the outset of the study, participants in the idiopathic infertility group were categorized according to WHO (2010) guidelines into ‘oligozoospermic’, ‘asthenozoospermic’, and/or ‘teratozoospermic based on the results of the initial semen analysis. Following antioxidant treatment, the number of participants in these groups showing an improvement in sperm quality was significantly improved. In a recent study, the same antioxidant formulation has been shown to increase the expression of mitochondrial proteins such as NDUSF1 to counteract oxidative stress [40]. Furthermore, the antioxidants were able to modulate the cellular pathways by the activation of the CREM signaling pathway, as well as inhibit the hypoxia and oxidative stress pathway and the protein oxidation pathway, all of which are deleterious for the spermatozoa [40]. This clearly indicates that antioxidant supplementation reduces seminal oxidative stress, which in turn has a beneficial effect on semen quality, which was observed in the current study.

Sperm DNA damage is one of the major consequences of continuous seminal oxidative stress [48]. Increased levels of seminal ROS result in post-testicular DNA damage [7]. Oxidative stress mediated DNA damage can be either directly because of oxidation of DNA base pairs, breaks in the DNA, and chromatin decondensation, or indirectly as a result of activating caspases and endonucleases. A strong correlation exists between high levels of ROS and SDF in infertile men [49,50]. Ultimately, SDF has an adverse effect on the fertilizing ability of spermatozoa. Simon et al. reported a low fertilization rate in patients with high levels of SDF using Comet assay [51]. Several studies have reported a negative correlation between SDF and fertilization rates, pregnancy, and embryo quality [52,53,54]. Barehet al. reported high incidence of SDF in normozoospermic male partners of couples with recurrent pregnancy loss (RPL) [55]. In the current study, we also noticed high levels of DNA damage in idiopathic infertile men, which decreased significantly following antioxidant supplementation with FH PRO for Men. Bio-availability of specific antioxidant components such as carnitine, arginine, Co-Q10, selenium, and vitamin E and C in recommended dosage could be the reason for the efficacy of FH PRO for Men. Previous studies have also discussed the beneficial effect of individual or combined oral antioxidants on reducing SDF levels in several male infertility conditions [19,56,57]. In the current study, the SDF levels in UMI patients decreased after antioxidant treatment, but this improvement in SDF did not reach statistical significance. Recently, Agarwal et al. demonstrated that the sperm proteins associated with cellular pathways such as cell death, necrosis, and apoptosis were deactivated in infertile men after treatment with FH PRO for Men [40]. Decreased SDF levels following treatment with FH PRO for Men indicate that the current results corroborate the proteomic findings.

The current study was prospective in design and not a double-blind or placebo-controlled clinical trial. Nevertheless, we recruited a total of 148 men in this internally controlled pilot study with the same subjects being examined before and after antioxidant treatment (FH PRO for Men). This had the advantage that we could identify the responders for each parameter that was tested. With this study design, where we had matched pairs of subjects, we could use the McNemar test to assess the efficacy of the antioxidant treatment. In the group of men with idiopathic infertility, specifically those with high ORP before the treatment, the McNemar test was significant, indicating the effectiveness of the treatment. By testing this way, we were then able to distinguish the efficacy of the treatment in idiopathic infertile men compared with patients with UMI, where the treatment did not show statistically significant results. This study focused mainly on the seminal and sperm parameters such as motility, normal morphology, sperm DNA fragmentation, and ORP. A lack of embryological results and complete pregnancy outcome is one of the limitations of this study.

In conclusion, in this internally controlled pilot study, we investigated a combination antioxidant formulation to treat MOSI. As the physiological antioxidant defense system is multi-layered and synergistically working, supplementation with a single vitamin or a formulation that does not support these synergistic functions may not be effective. In our case, we used FH PRO for Men, a balanced combination of various vitamins, essential co-factors for important antioxidant enzymes such as zinc, copper, and manganese for superoxide dismutase. It also contained selenium, a co-factor for glutathione peroxidase; powerful antioxidants such as lycopene and grape seed extract; and l-carnitine, a non-proteinogenic amino acid, essential for normal mitochondrial function as it shuttles fatty acids into mitochondria. The treatment was successful in the idiopathic group, resulting in a statistically significant improvement in sperm concentration, total motility, progressive motility, normal morphology, SDF, and ORP.

The results of our study add to the body of literature that aims to evaluate the effectiveness of treatment of male infertility with antioxidants. As discussed above, previous clinical trials have produced mixed results, and there is not yet consensus on the benefits of antioxidant treatment for male infertility. We believe that patient selection was a key factor in eliciting the beneficial effects of antioxidant supplementation with FH PRO for Men. As opposed to the majority of published studies on antioxidant supplementation, our clinical trial included patients with idiopathic male infertility and high seminal oxidative stress (91%; 108/119). In addition, the strongly favorable results observed in the current study suggest that perhaps a multi-ingredient formulation containing several different antioxidants, along with vitamins and minerals that serve as cofactors, may be superior to other antioxidant formulations that contain only a small number of nutrients, or that contain too low of a dose of the key antioxidants. The results of this study are encouraging, however, large-scale randomized, placebo-controlled trials that include embryological parameters such as fertilization, pregnancy, and live birth rates should be conducted.

## Figures and Tables

**Table 1:** Baseline summary statistics of idiopathic (at least one abnormal sperm parameter) and unexplained (normozoospermic) male infertility subjects.

Parameters	Idiopathic Male Infertility	*n*	Unexplained Male Infertility	*n*
Age (years)	36.4 ± 6.9 (36.0; 31.0–41.0)	119	34.0 ± 4.8 (34.0; 31.0–36.0)	29
BMI (Kg/m^2^)	29.6 ± 4.6 (29.4; 26.7–32.9)	119	29.7 ± 4.5 (28.7; 26.3–31.0)	29
Right testis size (mL)	14.2 ± 4.9 (13.1; 10.5–7.0)	87	16.1 ± 4.6 (16.5; 13.5–19.1)	24
Left testis size (mL)	13.8 ± 4.5 (14.0; 11.0–6.5)	87	15.3 ± 5.3 (13.9; 12.0–18.1)	24
Wife’s age (years)	31.6 ± 6.6 (31.0; 27.0–37.8)	119	27.9 ± 3.6 (27.0; 25.8–30.3)	29
Semen volume (mL)	3.1 ± 1.6 (3.0; 1.6–4.0)	119	3.0 ± 1.2 (3.0; 2.0–4.0)	29
Sperm concentration (10^6^/mL)	16.4 ± 17. 1 (11.0; 3.6–24.8)	119	46.2 ± 34.3 (36.0; 24.3–53.3)	29
Sperm viability (%)	48.6 ± 17.7 (50.0; 36.0–60.0)	76	N/A	
Total motility (%)	30.2 ± 16.3 (30.0; 16.3–40.0)	119	52.5 ± 8.3 (50.0; 45.0–60.0)	29
Progressive motility (%)	2.3 ± 5.5 (0.0; 0.0–0.0)	119	10.9 ± 9.9 (10; 0.0–20.0)	29
Normal morphology (%)	2.2 ± 1.7 (2.0; 1.0–3.0)	119	5.7 ± 2.5 (4.0; 4.0–7.0)	29
SDF (%)	43.5 ± 22.8 (42.0; 25.0–60.0)	83	28.2 ± 17.5 (21.0; 14.5–47.3)	29
ORP (mV/10^6^ sperm/mL)	12.4 ± 16.8 (5.5; 2.6–13.4)	119	1.6 ± 1.1 (1.3; 0.8–2.3)	29

All the values are presented as mean ± SD (median; interquartile range). BMI: body mass index, SDF: sperm DNA fragmentation, ORP: oxidation-reduction potential.

**Table 2:** Summary statistics of semen parameters, SDF, and ORP before and after the treatment of idiopathic infertile patients with FH PRO for Men.

Parameters	Before Treatment	*n*	After Treatment	*n*	Percent Change (Mean ± SEM)	*p* Value Wilcoxon Test
Semen volume (mL)	3.1 ± 1.6 (3.0; 1.6–4.0)	119	3.0 ± 1.4 (3.0; 2.0–3.5)	119	8.3 ± 3.9	0.9055
Sperm concentration (10^6^/mL)	16.4 ± 17.1 (11.0; 3.6–24.8)	119	25.5 ± 24.7 (17.0; 6.1–41.5)	119	141.1 ± 35.8	<0.0001
Sperm viability (%)	48.6 ± 17.7 (50.0; 36.0–60.0)	76	50.0 ± 18.2 (52.0; 43.3–61.3)	49	15.8 ± 9.3	0.4812
Total motility (%)	30.2 ± 16.3 (30.0; 16.3–40.0)	119	35.1 ± 18.9 (40.0; 16.3–50.0)	119	49.6 ± 13.9	0.0014
Progressive motility (%)	2.3 ± 5.5 (0.0; 0.0–0.0)	119	5.6 ± 7.8 (0.0; 0.0–10.0)	119	31.4 ± 6.7	<0.0001
Normal morphology (%)	2.2 ± 1.7 (2.0; 1.0–3.0)	119	4.1 ± 9.3 (3.0; 1.0–4.8)	119	96.3 ± 22.5	<0.0001
SDF (%)	43.5 ± 22.8 (42.0; 25.0–60.0)	83	34.3 ± 19.4 (28.0; 20.0–43.0)	83	−5.1 ± 6.4	0.0017
ORP (mV/10^6^ sperm/mL)	12.4 ± 16.8 (5.5; 2.6–13.4)	119	7.4 ± 15.7 (2.2; 0.9–6.2)	119	−25.3 ± 10.9	<0.0001

All the values are presented as mean ± SD (median; interquartile range). SDF: sperm DNA fragmentation, ORP: oxidation-reduction potential.

**Table 3:** Summary statistics of semen parameters, SDF, and ORP before and after the treatment with FH PRO for Men in idiopathic infertile patients having high ORP levels.

Parameters	Before Treatment	*n*	After Treatment	*n*	Percent Change (Mean ± SEM)	*p* Value Wilcoxon Test
Sperm concentration (10^6^/mL)	14.1 ± 15.6 (10.0; 3.1–19.0)	108	23.1 ± 22.9 (14.0; 5.4–38.0)	108	151.30 ± 39.3	<0.0001
Total motility (%)	30.2 ± 16.6 (30.0; 17.5–42.5)	108	34.9 ± 19.4 (40.0; 15.0–50.0)	108	51.3 ± 15.3	0.0043
Progressive motility (%)	2.5 ± 5.7 (0.0; 0.0–0.0)	108	5.7 ± 7.9 (0.0; 0.0–10.0)	108	28.9 ± 7.2	0.0002
Normal morphology (%)	2.2 ± 1.7 (2.0; 1.0–3.0)	108	4.1 ± 9.7 (3.0; 1.0–4.5)	108	92.1 ± 23.3	<0.0001
SDF (%)	43.0 ± 21.9 (42.5; 25.0–61.5)	67	37.1 ± 19.8 (30.0; 22.0–44.3)	67	−1.4 ± 7.2	0.0105
ORP (mV/10^6^ sperm/mL)	13.5 ± 17.2 (6.5; 3.6–14.5)	108	8.0 ± 16.4 (2.5; 1.1–8.6)	108	−27.3 ± 11.9	<0.0001

ORP ≥ 1.34 mV/10^6^ sperm/mL is considered as high. SDF: sperm DNA fragmentation, ORP: oxidation-reduction potential.

**Table 4:** Summary statistics of semen parameters, SDF, and ORP before and after the treatment with FH PRO for Men in idiopathic infertile patients having high SDF levels.

Parameters	Before Treatment	*n*	After Treatment	*n*	Percent Change (Mean ± SEM)	*p* Value Wilcoxon Test
Sperm concentration (10^6^/mL)	21.1 ± 16.9 (14.0; 11.0–27.0)	51	32.9 ± 28.2 (27.0; 13.0-44.3)	51	114.1 ± 47.0	0.0001
Total motility (%)	25.5 ± 12.7 (25.0; 15.0–30.0)	51	32.5 ± 18.1 (30.0; 16.3–50.0)	51	48.2 ± 16.5	0.0061
Progressive motility (%)	0.5 ± 1.8 (0.0; 0.0–0.0)	51	5.5 ± 8.0 (0.0; 0.0–10.0)	51	48.0 ± 11.5	0.0001
Normal morphology (%)	1.8 ± 1.2 (2.0; 1.0–2.0)	51	2.9 ± 2.3 (2.0; 1.0–4.0)	51	89.1 ± 20.8	0.0015
SDF (%)	56.5 ± 16.9 (55.0; 43.0–70.0)	47	42.4 ± 21.2 (39.0; 27.3–56.0)	47	−24.2 ± 4.7	<0.0001
ORP (mV/10^6^ sperm/mL)	6.1 ± 6.5 (4.2; 2.5–6.9)	51	3.4 ± 8.4 (1.7; 0.9–2.9)	51	−24.8 ± 21.1	<0.0001

SDF >30% is considered as high. SDF: sperm DNA fragmentation, ORP: oxidation-reduction potential.

**Table 5:** Summary statistics of semen parameters, SDF, and ORP before and after the treatment with FH PRO for Men in idiopathic infertile patients having high ORP and SDF levels.

Parameters	Before Treatment	*n*	After Treatment	*n*	Percent Change (Mean ± SEM)	*p* Value Wilcoxon Test
Sperm concentration (10^6^/mL)	19.9 ± 16.8 (13.0; 10.0–24.0)	46	30.4 ± 25.9 (24.5; 12.0–42.0)	46	119.6 ± 1.9	0.0008
Total motility (%)	25.4 ± 12.8 (25.0; 15.0–30.0)	46	32.7 ± 18.8 (32.5; 15.0–50.0)	46	53.0 ± 18.5	0.0089
Progressive motility (%)	0.5 ± 1.9 (0.0; 0.0–0.0)	46	5.7 ± 8.2 (0.0; 0.0–10.0)	46	48.9 ± 12.5	0.0002
Normal morphology (%)	1.8 ± 1.2 (2.0; 1.0–2.0)	46	2.9 ± 2.1 (3.0; 1.0–4.0)	46	88.6 ± 21.7	0.0026
SDF (%)	56.3 ± 16.5 (54.0; 43.0–70.0)	42	44.0 ± 20.3 (40.0; 28.0–59.0)	42	−20.5 ± 4.8	0.0002
ORP (mV/10^6^ sperm/mL)	6.7 ± 6.6 (4.4; 2.8–7.5)	46	3.7 ± 8.9 (1.9; 0.9–3.0)	46	−24.8 ± 23.3	<0.0001

ORP ≥1.34 mV/10^6^ sperm/mL and SDF >30% are considered as high. SDF: sperm DNA fragmentation, ORP: oxidation-reduction potential.

**Table 6:** Summary statistics of semen parameters, SDF, and ORP before and after the treatment of unexplained male infertility patients with FH PRO for Men.

Parameters	Before Treatment (*n* = 29)	After Treatment (*n* = 29)	Percent Change (Mean ± SEM)	*p* Value Wilcoxon Test
Semen volume (mL)	3.0 ± 1.2 (3.0; 2.0–4.0)	3.1 ± 1.2 (3.0; 2.0–4.0)	13.6 ± 8.8	0.4852
Sperm concentration (10^6^/mL)	46.2 ± 34.3 (36.0; 24.3–53.3)	51.5 ± 28.9 (52.0; 29.5–65.2)	29.4 ± 12.4	0.1872
Total motility (%)	52.5 ± 8.3 (50.0; 45.0–60.0)	52.2 ± 9.7 (52.0; 45.0–60.5)	0.6 ± 3.7	0.6495
Progressive motility (%)	10.9 ± 9.9 (10; 0.0–20.0)	17.9 ± 11.5 (15.0; 10.0–32.0)	46.3 ± 15.2	0.0024
Normal morphology (%)	5.7 ± 2.5 (4.0; 4.0–7.0)	6.7 ± 3.0 (6.0; 4.0–9.3)	31.4 ± 13.9	0.1779
SDF (%)	28.2 ± 17.5 (21.0; 14.5–47.3)	23.0 ± 11.6 (20.0; 15.8–28.3)	−5.9 ± 8.3	0.0306
ORP (mV/10^6^ sperm/mL)	1.6 ± 1.1 (1.3; 0.8–2.3)	1.1 ± 0.9 (0.9; 0.5–1.4)	−19.6 ± 11.7	0.0168

All the values are presented as mean ± SD (median; interquartile range). SDF: sperm DNA fragmentation, ORP: oxidation-reduction potential.

**Table 7:** Effect of FH PRO for Men on seminal ORP and SDF in men with idiopathic male infertility and unexplained male infertility.

Group	Category	Number of Patients (Percentage)		
		Before Treatment	After Treatment	*p*-Value McNemar Test
Idiopathic male infertility	High ORP ^a^	108 (90.8%)	75 (63.0%)	<0.0001
	High SDF ^b^	47 (60.3%)	35 (44.9%)	0.0227
Unexplained male infertility	High ORP ^a^	14 (50.0%)	8 (28.6%)	0.1796
	High SDF ^b^	10 (34.5%)	5 (17.2%)	0.0625

^a^ Cut-off value for ORP = 1.34 mV/10^6^ sperm/mL, ^b^ cut-off value for SDF = 30%. SDF: sperm DNA fragmentation, ORP: oxidation-reduction potential.

## Supplementary Materials

The following are available online at https://www.mdpi.com/2076-3921/9/3/219/s1, Table S1: Number and percentage of patients with oligozoospermia, asthenozoospermia and teratozoospermia before and after the treatment with FH PRO for Men. Total number of patients included in the study: *n* = 119.
