# Supplementary material for: Efficacy of Antioxidant Supplementation on Conventional and Advanced Sperm Function Tests in Patients with Idiopathic Male Infertility
Source: Antioxidants (Basel). 2020 Mar 6;9(3):219. doi: 10.3390/antiox9030219 (PMC7139646; doi:10.3390/antiox9030219)
Supplement: Supplementary file 1 [file antioxidants-09-00219-s001.pdf]

Supplementary Materials:

Table S1. Number and percentage of patients with oligozoospermia, asthenozoospermia and teratozoospermia before and after the treatment with FH PRO for Men. Total number of patients included in the study: $n = 119$.

**Group**	**Number of Patients (Percentage)**			
	**Before Treatment**	**After Treatment**	**Improvement**	p-value McNemar Test
Oligozoospermic	79 (53.4%)	56 (37.8%)	45 (56.9%)	0.0026
Asthenozoospermic	72 (48.6%)	53 (35.8%)	29 (40.3%)	0.0064
Teratozoospermic	98 (66.2%)	70 (47.3%)	33 (33.7%)	<0.0001
